# Virulence genes contributing to *Aeromonas veronii* pathogenicity in Nile tilapia (*Oreochromis niloticus*): approaching the development of live and inactivated vaccines

**DOI:** 10.1007/s10499-022-01023-1

**Published:** 2022-11-19

**Authors:** Hadeer A. Youssef, Hala F. Ayoub, Eman I. Soror, Aya F. Matter

**Affiliations:** 1 Department of Aquatic Animals Medicine, Faculty of Veterinary Medicine, MoshtohorBenha University, Benha, Egypt; 2 Department of Fish Health and Management, Central Laboratory for Aquaculture Research (CLAR) Agricultural Research Center (ARC), Abbassa, Sharqia Egypt

**Keywords:** *Aeromonas veronii*, Live vaccine, Pathogenicity, Virulence genes

## Abstract

This study aimed to develop and evaluate live and inactivated vaccines to *Aeromonas veronii* pathogenicity in Nile tilapia. Therefore, five well-identified *Aeromonas veronii* isolates, including A (HY1), A (HY2), A (HY3), A (HY4), and A (HY6) isolated from diseased Nile tilapia (*Oreochromis niloticus*), were used for vaccine preparation. Virulence genes detected by a polymerase chain reaction (PCR) and lethal dose determination were conducted. Nile tilapia, each with a body weight of 25 ± 0.5 g were divided into six experimental groups (each of 20): T1 group (control), fish were injected with saline as a negative control, T2 group (formalin-killed vaccine) for the A (HY2) strain, T3 group ( formalized killed vaccine) for the A (HY4), T4 group (autoclaved vaccine) for the A (HY2), T5 group (autoclaved vaccine) for A (HY4), and T6 (live vaccine) for A (HY1), triplicate. At the end of the immunization period, all groups were challenged by *A. veronii*, A (HY2). Blood samples were drawn 21 days post-immunization and 3 days after the challenge test for antibody titer assay. The results showed that the pathogenicity of strains A (HY2) and A (HY4) was the strongest, as the lethality rates (LR) were 100% and 90%, respectively, whereas the pathogenicity was moderate for strains A (HY3) and A (HY6) (LR 60% for each). A (AY1) was the weakest strain as no dead fish was found for this strain. The presence of *alt*, *act, aerolysin, lipase,* and *fla* genes as the main cause of the pathogenesis. The best protective efficacy was obtained from the live vaccine, A (HY1) with a protective rate of about 94.12% (relative percentage of survival, RPS), compared to autoclaved killed vaccines and formalin-killed vaccines. Based on immunoglobulin estimation (IgM) and RPS%, our data concluded that A (HY1) live vaccine had the best vaccine prophylactic effect against the highly pathogenic strain A(HY2).

## Introduction

Bacterial diseases caused by motile aeromonads represent the most common problems in aquaculture freshwater fish. They are considered the body of knowledge that generally links *Aeromonas hydrophila and A. veronii* infection (Austin and Austin 2016; Dong et al. 2017a, 2017b). *Aeromonas hydrophila* and other motile *Aeromonas* species, including *A. sobria*, *A. veronii*, and *A. cavieae* are pathogens that cause motile *Aeromonas* septicemia and are also linked to public health risks in humans (Janda and Abbott 2010). *Aeromonas veronii* belongs to the *Aeromonadaceae* family and it is a Gram-negative, rod-shaped, mesophilic, motile bacterium. Furthermore, it can be found in a variety of aquatic settings (Parte 2014).

Previous research shows that many number of interconnected factors cause the pathogenicity of *Aeromonas*. There are confirmed virulence factors, such as outer membrane proteins, toxins, proteases, motility-related factors, secretion systems, quorum, and iron ion acquisition systems, as well as hemolytic and cytotoxic activities (Qin et al. 2022). Pathogenicity was related to the presence of virulence genes in *Aeromonas* strains, which play an essential role in disease development (Hossain et al. 2018; Sun et al. 2016; Zhang et al. 2019). Antibiotics are used to control bacterial diseases in aquaculture. However, this can lead to the development of antibiotic resistance and antimicrobial residues in aquaculture (Vivekanandhan et al. 2002). A recent study by Salah et al. (2021) demonstrated a multi-drug resistance of *Aeromonas hydophila* chloramphenicol (67.4%), followed by amikacin (51.9%) and gentamicin (47.1%).

Many researchers have developed vaccines against *A. hydrophila*, primarily in warm water fishes. The experimental vaccine can protect many fish species from *A. hydrophila* infection following vaccination (Aoki 1999). The commercial vaccine efficacy is complicated by many phenotypic and serological specificities found in *Aeromonas* spp. (Nielsen et al. 2001; Toranzo et al. 2009). Vaccines are formulated using either antigens (pathogenic bacterial), or whole bacterial killed cells to activate the innate and the cellular and humeral immune (Adams 2019) The effectiveness of a good vaccine is related to the appropriate immunization routes for stimulating where intraperitoneal (i.p.) injection has mostly provided good results, although it is stressful to the animals, impractical for farmer level, labor intensive, and expensive (Assefa and Abunna 2018). Another route of immunization is recently used by feed-based oral immunization that proved to be less laborious, more applicable for mass vaccination at farmer level (Monir et al. 2020). There are different types of vaccines used in tilapia including inactivated monovalent vaccine, inactivated polyvalent vaccine, live bacterial vaccine, and recombinant vaccines ( Shirajum Monir et al. 2020).

Nile tilapia (*Oreochromis niloticus*) comprise a well-known freshwater fish genus that includes one of the world’s most popular cultivated fish kinds. They are cultivated in many countries ranging from extensive to intensive/commercial ponds. It is the world’s second-largest group of cultured freshwater fish (Mapenzi and Mmochi 2016). Although tilapia aquaculture has improved rapidly, it still faces enormous problems from the number of devastating bacterial and viral diseases (Austin and Austin 2016).

Six virulence genes related to pathogenicity including aerolysin, cytotonic enterotoxins, elastase, glycerophospholipid: cholesterol acyltransferase, lipase, and serine protease were identified *A. veronii* isolate from diseased crucian carp (*Carassius auratus gibelio*) (chen et al 2019). Moreover, The prevalence of the virulence genes detected in the isolated motile aeromonads from tilapia was aerolysin (aer), 52.2%; elastase (ahp), 26.25%; hemolysin (hyl), 35%; and lipase (lip), 3.75% (El-Gohary et al. 2013).

Vaccines for aquaculture have been successful in reducing the use of antibiotics, especially in developed countries (Sommerset et al. 2005; Gudding and Van Muiswinkel 2013). The use of antibiotics has obvious drawbacks, more predominantly, the risk to public health because of the development of antimicrobial resistance. In order to reduce the use of chemicals and antibiotics in aquaculture and marine environment, the use of vaccination is suggested (Adams 2019).

In this study, different kinds of *Aeromonas veronii* vaccines have been developed and their efficacy was tested in *O. niloticus* Nile tilapia. The virulence genes were detected using PCR to develop live and inactivated vaccines. Their efficacy was determined by measuring antibody titers in fish sera.

## Materials and methods

### Bacterial strains

Five well-identified *Aeromonas veronii* isolates (A (HY1), A (HY2), A (HY3), A (HY4), and A (HY6) were isolated from diseased fish as previously (Youssuf et al. 2020). The accession number of *A. veronii* isolates is represented in Table 1. PCR products were purified using QIA quick PCR Purification kit (QIAGEN, USA) following the manufacturer’s protocol. Then the purified product was sequenced in the forward and/or reverse directions on an Applied Biosystems 3130 automated DNA Sequencer (ABI3130, USA) using a ready reaction Bigdye Terminator V3.1 cycle sequencing kit (Cat. No. 4336817, Perkin- Elmer/Applied Biosystems, Foster City, CA). The resultant sequenced Aeromonas strains were analyzed using the National Center for Biotechnology Information (NCBI), Basic Local Alignment Search Tool (BLAST) program, and the neighbor-joining blast tree against the database of strain types (the most prevalent and pathogenic Aeromonas isolates) and published valid prokaryotic nomenclature (Youssuf et al. 2020).

**Table 1:** Accession numbers of *A. veronii* isolates

Samples	NCBI accession number
*A* (*HY2*)	MK584926 *A. veronii* HY2
*A* (*HY1*)	MK584925 *A. veronii* HY1
*A* (*HY3*)	MK584927 *A. veronii* HY3
*A* (*HY4*)	MK584928 *A. veronii* HY4
*A* (*HY6*)	MK584930 *A. veronii* HY6

### PCR detection of virulence genes

#### DNA extraction

A loopful from gills, liver, kidneys, and spleen of the clinically diseased fish were inoculated into Tryptic soy broth (TSB, Oxoid, UK) then streaked over Tryptic soy agar (TSA, Oxoid, UK), incubated for 24 h at 28 °C (Austin and Austin 2016). Pure colonies were stored at − 80 °C in TSB containing 20% glycerol for further studies following Youssuf et al. (2020).

The QIAamp DNA Mini kit (Qiagen, Germany, GmbH) was used to extract bacterial DNA following the manufacturer’s recommendations, with some modifications. First, 200-µl bacterial sample suspension was treated at 56 °C for 10 min in a 10-µl proteinase K and 200-µl lysis buffer mixture. After the incubation, 200-µl ethanol (100%) was added to the lysate. Then, nucleic acid was eluted using the 100-µl elution buffer that was provided in the kit. Finally, the sample was washed and centrifuged following the manufacturer’s recommendations.

#### PCR detection of virulence genes

The Primers used were supplied by Metabion (Germany) and represented in Table 2. They were used in a 25-µl reaction that included 12.5-µl Emerald Amp Max PCR Master Mix (Takara, Japan), 1 µl each primer of 20-pmol concentration, 5.5-µl water, and 5-µl DNA template. The reaction was performed in an Applied Biosystem 2720 thermal cycle conditions and was adjusted for each primer according to the reference ( Table 2).

**Table 2:** Primers sequences, target genes, amplicon sizes, and cycling conditions for conventional PCR

Target gene	Primer sequences	Amplified segment (bp)	Primary denaturation	Amplification (35 cycles)			Final extension	Reference
				**Secondary denaturation**	**Annealing**	**Extension**		
*Aerolysin* (encodes hemolytic toxin)	CACAGCCAATATGTCGGTGAAG	326 bp	94 °C 5 min	94 °C 30 s	52 °C 40 s	72 °C 40 s	72 °C 10 min	Singh et al. (2008)
	GTCACCTTCTCGCTCAGGC							
*Act* (encodes cytolytic enterotoxin)	AGAAGGTGACCACCACCAAGAACA	232 bp	94 °C 5 min	94 °C 30 s	55 °C 30 s	72 °C 30 s	72 °C 7 min	Nawaz et al. (2010)
	AACTGACATCGGCCTTGAACTC							
*Ast* (encodes enterotoxin)	TCTCCATGCTTCCCTTCCACT	331 bp	94 °C 5 min	94 °C 30 s	55 °C 30 s	72 °C 30 s	72 °C 7 min	
	GTGTAGGGATTGAAGAAGCCG							
*Alt* (encodes extracellular lipase)	TGACCCAGTCCTGGCACGGC	442 bp	94 °C 5 min	94 °C 30 s	55 °C 40 s	72 °C 40 s	72 °C 10 min	
	GGTGATCGATCACCACCAGC							
*Fla* (encodes flagella subunit protein)	TCCAACCGTYTGACCTC	608 bp	94 °C 5 min	94 °C 30 s	55 °C 40 s	72 °C 45 s	72 °C 10 min	
	GMYTGGTTGCGRATGGT							
*Lipase*	ATCTTCTCCGACTGGTTCGG	382 bp	94 °C 5 min	94 °C 30 s	55 °C 40 s	72 °C 40 s	72 °C 10 min	Sen and Rodgers 2004
	CCGTGCCAGGACTGGGTCTT							

#### PCR product analysis

PCR products were separated using electrophoresis on 1.5% agarose gel (Applichem, Germany, GmbH) in 1 × TBE buffer at room temperature using gradients of 5 V/cm. For gel analysis, 20-µl PCR products were loaded in each gel slot. The fragment sizes were determined by Generuler 100 bp ladder (Qiagen, Gmbh, Germany). A gel documentation system (Alpha Innotech, Biometra) was used to photograph the gel, and the data was processed using Bio Doc Analyze Analysis Software (BDA).

### Lethal dose determination

One hundred eighty healthy Nile tilapia with an average weight of 25 ± 0.5 g were acclimated for 14 days at a temperature of 25 ± 1 °C. Fish were randomly divided into six groups, ten fish in triplicate. *A. veronii* isolates were inoculated into (BHI) broth and grown overnight at 28 °C. Bacterial cells were obtained by centrifugation then adjusted by spectrophotometer apparatus at OD_600_ of 0.6 using sterile 0.85% NaCl (saline solution). In these conditions, the number of bacteria was about 5 × 10^8^ CFU/ml. To resolve the pathogenicity of isolates, *O. niloticus* were injected with 0.1-ml bacteria ranging from 5 × 10^4^ to 5 × 10^7^ CFU/fish. These bacterial suspensions were prepared through a series of fivefold serial dilution steps. For each *A. veronii* isolate, five groups (150 fish) were injected, except the control group that was injected with a sterile saline solution. All groups were monitored for 7 days and the dead fish were recorded every 12 h. The LD50 was determined following Hodson et al. (1984). 1 × 10^7^ CFU/fish bacterial dose in the challenge test was used to confirm the pathogenicity of the isolates. *O. niloticus* were monitored every 12 h for 7 days, and the lethality rate (LR) was calculated as previously described (Bailonea et al. 2010). Random samples of fish were sacrificed and examined for parasites, mycotic, and bacterial pathogens as described by Austin and Austin (2007) to ensure their normal health status.

### Experimental design and vaccine preparation

*A. veronii* vaccines (inactivated and live) were fabricated as described by Li et al. (2011). Briefly, formalin-kill vaccine (FKV) from A (HY2) and A (HY4) strains were cultivated until they reached an OD600 of 1.0, then the bacteria were harvested to produce an inactivated vaccine in 0.5% formalin. Cells were centrifuged, washed three times, and resuspended in sterile saline solution after 24-h incubation at 37 °C. Autoclaved-kill vaccine (AKV) from the same previous strains was prepared by autoclaving the bacterial culture in the nutrient broth in the autoclave at 121 °C for 15 min. The treated culture was centrifuged at 7000 × g for 30 min. Cell pellets were washed twice using PBS and resuspended again in PBS following Bactol et al. (2018). Strain A (HY1) that was chosen to produce the live vaccine was diluted using sterile saline solution until an OD_600_ of 0.2 to adjust 1 × 10^8^ CFU/ml of bacterial concentration. We used this strain A (HY1) because it is a naturally attenuated strain where no mortalities were recorded in the control group and *A. veronii* HY1 group throughout the experimental period in the previous study (Youssuf et al. 2020). All the procedures were performed under sterile conditions. Vaccines were kept at 4 °C until further use. These vaccines were subjected to sterility and safety tests as previously conducted (Anderson et al. 1970).

## Experimental design and vaccination

The experiment included six groups as shown in the following table:

**Table:** 

Group	Treatment	Strains
T1 group	Control (not vaccinated)	
T2 group	Formalized killed vaccine	A (HY2)
T3 group	Formalized killed vaccine	A (HY4)
T4 group	Autoclave killed vaccine	A (HY2)
T5 group	Autoclave killed vaccine	A (HY4)
T6 group	Live vaccine	A (HY1)

To prevent cross-infection, each group was kept in a separate tank for the trial duration. For 3 weeks, fish were fed at a rate of 5% body weight twice daily (at 9:00 and 17:00). Feed amounts were adjusted bi-weekly to fit the new fish biomass. Every day, fish waste was drained and 50% of the water was replaced with aerated and dechlorinated water from the storage tank. Groups T2, T3, T4, T5, and T6 received 0.1 ml from the prepared vaccines as described previously (Surgha et al. 2021). The T1 control group received a sterile saline post-intraperitoneal injection (I/P) and stay as a negative control. The immunization period was extended for 21 days. The water temperature and dissolved oxygen (DO) were monitored twice a day at 8:00 and 16:00, while the other water quality parameters were measured once a week. Water temperature and DO were 26 ± 1 °C and 6 ± 0.2 mg/L, respectively. The pH value was 7.5 ± 0.5. All these parameters were within the optimum fish growth requirement.

### Challenge test

After 21 days post-vaccination, each fish (10 fish/duplicates), vaccinated and positive control was challenged by 0.1-ml intraperitoneal injection (I/P) of strain A (HY2), (1.0 × 10^8^ CFU/ml). Another 20 fish were randomly selected and injected with (1.0 × 10^8^ CFU/ml) of PBS and used as a negative control (NC) following Zhang et al. (2019), the typical signs of diseased fish were external hemorrhages and inflammation or ulcers in gills, fin base, and other tissues. Mortalities were renowned every 12 h for 7 days, and the cumulative mortality rate was determined. The relative percentage of survival (RPS) was calculated according to Amend (1981) as follows:

RPS = [1 − (mortality in vaccinated group/mortality in control group)] × 100%.

### Serum collection

Blood was obtained from three fish/replicates 21 days post-vaccination and 3 days post-challenge test. Three samples from each group post-vaccination and post-challenge test were obtained from each fish using a 1-mL syringe with a 25-G needle attached. The needle was inserted into caudal blood vessels. Collected blood was stored in Eppendorf tubes and was left overnight to clot. Clot bordering was done and centrifuged. The collected serum was preserved at − 20 °C until use.

### Immunoglobulin assay

Antibody titers were measured spectrophotometrically in fish sera following the protocol of ELISA kits (Cusabio Biotech Co. Ltd., USA). Briefly, 50 μl of standard or sample per well was added per well. Then 50 μl of HRP-conjugate mixed solution to each well (not to Blank. well) was added and mixed well and then incubated for 1 h at 37 °C. Each well was aspirated and washed, repeating the process two times for a total of three washes. The wash occurred by filling each well with wash buffer (200 μl) let it stand for 10 s. Complete removal of the liquid at each step is essential to good performance. After the last wash, any remaining wash buffer was removed by aspirating or decanting. The plate was then inverted and blotted against clean paper towels. Fifty μl of substrate A and 50 μl of substrate B were added to each well and mixed well. The plate was incubated for 15 min at 37 °C. Then 50 μl of stop solution was added to each well, and the plate was thoroughly mixed. The optical density was determined of each well within 10 min, using a microplate reader set to 450 nm.

### Statistical analysis

Statistical significance across groups was analyzed using one-way analysis of variance with post hoc Tukey’s tests, using SPSS 20.0 software to determine the significant changes in immunoglobulin levels 21 days post-immunization and 3 days post-challenge with A (HY1). A *p*-value < 0.05 was considered significant.

## Results

### Virulence genes of Aeromonas veronii

Among the *Aeromonas* isolates, DNA analysis showed five *A. vernoii* strains. PCR was used to detect virulence factors in previous isolates. The results indicated that they have many genotypes.

*Fla* genes were found in all strains. *Act* and *alt* genes were also found in all strains except A (HY1) for *act* and A(HY6) for alt. *lipase* gene were found in three strains, while *aerolysin* gene was detected in all strains except A (HY3) and A (HY6). The *ast* gene was absent in all strains (Table 3).

**Table 3:** Virulence genes, pathogenicity test, and lethality rate of *Aeromonas veronii*

Sample	*Aerolysin*	*act*	*alt*	*ast*	*Fla*	*lipase*	Results of pathogenicity test	NCBI accession number
*A* (*HY2*)	+	+	+	-	+	+	**100%**	MK584926 *A.veronii* HY2
*A* (*HY1*)	-	-	+	-	+	-	**0%**	MK584925 *A.veronii* HY1
*A* (*HY3*)	-	+	+	-	+	-	**60%**	MK584927 *A.veronii* HY3
*A* (*HY4*)	+	+	+	-	+	+	**90%**	MK584928 *A.veronii* HY4
*A* (*HY6*)	-	+	-	-	+	+	**60%**	MK584930 *A.veronii* HY6

100% is highly pathogenic

90% is moderate pathogenicity

60% is mild pathogenic

0% is not pathogenic

### Virulence genotype and pathogenicity

Previous research has shown that virulence factor genes are responsible for the pathogenicity of *A. veronii*. To investigate the relationship between virulence genotype and pathogenicity, the lethal dose (LD_50_) was determined using different bacterial cell concentrations for the five isolates. Virulence genes, pathogenicity test, and lethality rate (LR) results of *A. veronii* are represented in Table 3. The pathogenicity of strains A (HY2) and A (HY4) was the strongest, whereas the LRs were 100% and 90%, respectively and these strains explained expression for all genes except for *ast* gene only. The strains of A (HY3) and A (HY6) pathogenicity were moderate, whereas the LR was 60%, and these strains explained the expression for three virulent genes. A (HY1) was the weakest strain as the expression was only for two genes, and no dead fish was found for this strain. Because of the high pathogenicity of A (HY2) and A (HY4) strains, they were used for vaccine formulation.

### Immunoglobulin response for live and inactivated vaccines

After 21 days post-vaccination, the IgM level for live A (HY1) vaccine was the highest (59.29^a^ ± 2.97) followed by AKV for A (HY4) and A (HY2) (50.24^b^ ± 0.70), (45.65^bc^ ± 1.28) respectively. While the lowest levels of IgM were (44.29^c^ ± 0.26), (37.08^d^ ± 2.14) for FKV A (HY4), FKV A (HY2), respectively, they were still relatively high compared to the control group (Table 4). However, the result for IgM levels began to reduce 3 days post-challenge. Although it remained significantly higher (*p* < 0.05) than the control group. Also, IgM levels were high for live (HY1) vaccine groups (57.29^a^ ± 3.55) followed by AKV groups for A (HY4) and A (HY2) (46.80^b^ ± 3.04), (44.15^b^ ± 1.57), respectively. Also, the FKV for A (HY4), A (HY2) explained the low IgM level (36.65^c^ ± 0.66), (37.08^c^ ± 1.56) compared with other groups (Table 4).

**Table 4:** Immunoglobulin levels (µg/ml) for *O. niloticus* after 21 days post-immunization and 3 days post-challenge

Group	IgM 21 d post-immunization	IgM 3-days post challenge (µg/ml)
Control	33.85^d^ ± 0.37	33.60^c^ ± 0.20
F (HY4)	44.29^c^ ± 0.26	36.65^c^ ± 0.66
A (HY4)	50.24^b^ ± 0.70	46.80^b^ ± 3.04
F (HY2)	37.08^d^ ± 2.14	37.08^c^ ± 1.56
A (HY2)	45.65^bc^ ± 1.28	44.15^b^ ± 1.57
L (HY1)	59.29^a^ ± 2.97	57.29^a^ ± 3.55

Data are expressed as mean ± SE. Different superscript of the means of the same column means significant difference at *p* < 0.05

### Relative percentage of survival (RPS)

**Fig. 1:**
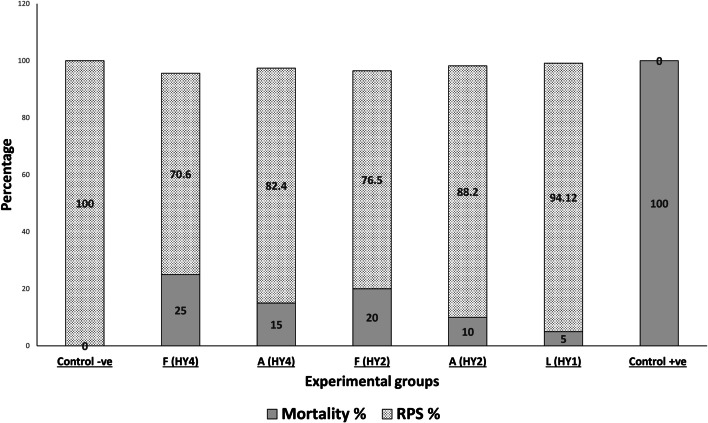
Mortality rate and the relative percentage of survival (RPS) of *O. niloticus* challenged by *A. veronii (*HY2*)* isolates

The RPS% of the live vaccine A (HY1) group was higher than the AAKV A (HY2), A(HY4) and higher than FKV (HY2) A (HY4). The mortality rate was 0% in the negative control group (Table 5, Fig. 1).

**Table 5:** Mortality rate and the relative percentage of survival (RPS) of *O. niloticus* challenged by *A. veronii (*HY2*)* isolates

Item	Control -ve	F (HY4)	A (HY4)	F (HY2)	A (HY2)	L (HY1)	Control + ve
Fish no	20	20	20	20	20	20	20
1st day	0	1	0	1	1	0	1
2nd day	0	2	2	1	0	1	3
3rd day	0	2	1	2	1	0	5
4th day	0	0	0	0	0	0	4
5th day	0	0	0	0	0	0	4
6th day	0	0	0	0	0	0	3
7th day	0	0	0	0	0	0	0
Mortality%	0	25	15	20	10	5	100
RPS%	100	70.6	82.4	76.5	88.2	94.12	0 .0

*F* means formalin-killed vaccine, *A* means autoclaved killed vaccine, *L* means live vaccine

## Discussion

Aeromonads have been identified as the causative agent of fish disease by researchers. The distribution of *Aeromonas* species in aquatic environments proposes that their interactions with fish are continual and inescapable, which most likely explains their opportunistic pathogenicity (Ottaviani et al. 2011). Over the past decade, Aeromonads, including *A. hydrophila* and *A. veronii* have been linked to fish mortality worldwide, and are considered the primary cause of fish outbreaks and causing massive economic losses (Janda and Abbott 2010; Noga 2010). Motile aeromonads are extremely diverse, and not only *A. hydrophila* can cause the disease but *A. veronii* and other *Aeromonas* species have also been recorded as a major threat to freshwater fish aquaculture (Dong et al. 2015; Zhu et al. 2016). Several virulence genes have been discovered in *A. veronii,* which help to explain its pathogenicity. Additionally, the detection of *aerolysin and alt* genes has recently been suggested as the main cause of virulence (Wang et al. 2003). Some virulence genes were discovered here in *A. veronii isolates* that were isolated from diseased fish. Reports of *A. veronii* in the USA and *A. hydrophila* in China demonstrated that *aerolysin* is the major contributor to the virulence of the pathogenic *Aeromonas* strains (Nawaz et al. 2010; Zheng et al. 2012a, b). Consistent with this, our results show *A. veronii* virulence does not arise from one gene, but it is probably the result of the collaborative effects of different virulence genes. Our investigation is accordant with the *alt* + , *act* + , and *fla* + genotypes are playing an essential role in *A. veronii* pathogenicity since A (HY2) and A (HY4) produced a tremendous mortality rate, with an average LD50 value of 5 × 10^6^ and 5 × 10^7^ CFU/fish.

Many studies have been targeted on the immunoprotective characteristics of secreted proteins, just as these consistently affect bacterial virulence (Zhang et al. 2014). For instance, in *A. hydrophila*, *hemolysin*, and *aerolysin* are extracellular proteins that are well-known virulence factors (wang et al. 2019). However, it was discovered that *aerolysin* is the most important gene for *A. veronii* virulence. In all the types of infections with *A. veronii*, signs such as hemorrhagic and ulcerative lesions on the skin and other organs are produced by aerolysin (aer)‐mediated aerotaxis (Bücker et al. 2011). The gene aer is a cytotoxic pore‐forming enterotoxin which was reported to be one of the major virulence factors in the pathogenesis of *A. veronii*‐associated fish diseases (Baumgartner et al. 2017; Zheng et al. 2012a, b). This study describes the protective capacity of a live vaccine for *A. veronii* in *O. niloticus* delivered through injection vaccination. Additionally, the immunization was tested by infecting fish with A(HY1) through intraperitoneal injection. The vaccines are administered to stimulate humoral immune responses (IgM) of immunized fish. The agglutination test is a simple and easy assay to perform that measures antibody amount in the serum of vaccinated hosts or organisms (Tizard 1996). The assessment of agglutinating antibody titer is an easy approach to measure circulating antibodies in serum samples collected from fish previously immunized with particulate antigen preparations (Sugahara and Eguchi 2012). In addition, Morrison and Nowak (2002) reported that the agglutination assay is considered quick, simple, and inexpensive among the serological tests. Blood extraction was drawn 21 days post-vaccination and 3 days post-challenge test from *O. niloticus*; the results of the study showed that vaccinated *O. niloticus* with live vaccine produced greater antibody levels in serum than non-vaccinated fish. Alternatively, immunoglobulin was elevated post-immunization and even after the challenge test. Similar to the results obtained from Zhang et al. (2017), who reported that immunized catfish (*Ictalurus punctatus*) with recombinant proteins significantly increased the relative percentage of survival (RPS), compared with the unimmunized catfish. Interestingly, the formalin-killed vaccine (FKV) results are consistent with Wang et al. (2014), who reported a remarkable increase in antibody (IgM) levels to post-intraperitoneal vaccination with an FKV against *S. iniae* infection in tilapia. An I/P infection route was used to infect the fish to ensure a systemic immune response from the oral vaccination. The vaccination routes have a significant effect on vaccine efficacy. Systemic immune responses are readily elicited by intramuscular intraperitoneal injections and provide better protection levels than oral routes. However, the oral route is easy and simple in its application (Grabowski et al. 2004). Although, this study demonstrated superior antigenicity of formalized and autoclaved vaccines.

These vaccines were prepared by attenuating live bacterial cells with physical methods, like formalin and autoclaving, which is alike to that of Dehghani et al. (2012), who documented that formalin-killed and heat-killed *A. hydrophila* vaccines are evenly antigenic in red trout. Nevertheless, this study revealed that formalin-killed whole-cell vaccine has lower antigenicity.

Additionally, the genotype *alt* − /*act* − /*fla* − /*lipase* − /*aerolysin* + exhibits loss of virulence and unharmed immunogenicity that could become potential candidates that are used as live vaccines for fish immunization. Anyhow, further studies are crucial to determine the efficacy of these alternatives across different *A. veronii* strains.

The overall results of the study paved the way for the future development of different types of vaccines to control the bacterial diseases hindering the aquaculture industry, particularly Aeromonas, as alternatives for the intensive use of antimicrobials that have side effects exceeding their therapeutic interventions. In addition, this study could represent the initial step to control the pseudomonas septicemia in the intensive tilapia culture in Egypt, where dependance on this species as a source of protein is a priority. However, the vaccine development should be carefully evaluated to properly assess its efficacy and monitor any inadvertent health effects on tilapia aquaculture.

## Conclusion

In conclusion, our study reports the link among virulence genotype, pathogenicity, and immunogenicity of *A. veronii* isolated from the diseased fish. Different genotypes were applied to identify novel antigens for new vaccine development. The live vaccine was the best against *A. veronii* infection for *O. niloticus* protection.

## Data Availability

The data that support the findings of this study are available from the corresponding author upon request.
